# Mr. P. J. Michelli

**Published:** 1906-05-26

**Authors:** 


### MR. P. J. MICHELLI

### AT HOME AT THE ROYAL VICTORIA AND ALBERT DOCKS HOSPITAL.

On the conclusion of his year of office as President of the Hospital Officers' Association, Mr. P. Michelli, the Secretary of the Seamen's Hospital, held an "At Home" at the Royal Victoria and Albert Docks Hospital on Saturday, which was attended by a large number of the executive officers of the London hospitals and others. Amongst those present were Sir Frederick Young, Sir Francis Lovell (Dean of the London School of Tropical Medicine), Mr. Keith D. Young (Architect to the Seamen's Hospital Society), and Mr. Pite (Architect to King's College Hospital).

A demonstration of microscopic specimens of the parasites of malaria, sleeping sickness, etc. was given by Dr. Stanton in the laboratories of the London School of Tropical Medicine, which has its headquarters at the hospital, whilst Dr. Daniels, the Medical Superintendent of the School, gave a most interesting magic-lantern exhibition in connection with tropical medicine in the theatre. Besides this the P. & O. and the Ocean S.S. Companies had each kindly thrown open one of their vessels to the inspection of the guests. Mr. Keith D. Young, the architect of the hospital, also conducted the guests during the course of the afternoon on a tour of inspection over the buildings.

The whole of the proceedings were well organised and a most instructive and enjoyable afternoon was passed. Mr. Michelli has proved himself a most active, able, and popular President, and his year of office will be memorable for its results in the history of the Association.

### The Tropical Hospital and School.

This branch of the Seamen's Hospital at Greenwich, which now contains fifty beds, and in the grounds of which has been erected the London Tropical School of Medicine, has grown up from very small beginnings. The original hospital was built in 1889, and contained only thirteen beds, an out-patients' waiting-room, with a combined consulting-room and dispensary, and resident quarters for one medical officer, a matron and six nurses, and four servants. Ten years later, in 1899, the first portion of the Medical School was erected, together with the new ward block of the hospital. This new ward block contains two bright and cheerful wards (86 feet long by 27 feet wide), providing accommodation for thirty-eight beds, with a ward scullery to each, and bath and lavatory accommodation in an adjoining tower. Above these wards on the top floor are twelve bedrooms and a day-room for the nurses and on the same floor is a well-fitted and excellently lighted operation theatre with a sterilising room adjoining. At the back of the hospital a small but well-equipped laundry, in which the washing of the parent hospital at Greenwich is done, has been built, with a high-pressure steam disinfecting apparatus attached. A new kitchen block connected with the hospital by a covered way, has been added between the old and new portions of the building in place of the small kitchen which supplied the original institution, and this block contains the kitchen, scullery, milk-store, larder, and porter's sitting-room.

On the left of the hospital is situated the Medical School block, consisting on the ground floor of a large laboratory (88 feet long by 17 feet wide), exceptionally well lighted and equipped, with a smaller laboratory for the Superintendent at the end. Leading off this is the Bobaji Petit Museum, whilst on the same floor are a mess-room and serving-room capable of seating forty students. Above, on the first floor, are the lecture-room and twelve bedrooms for students, and on the second floor are other bedrooms, another laboratory, and a store-room.

Adjoining the Medical School is the new out-patient department, comprising a general waiting-room, a consulting-room with an examining-room attached, dressing-room, and the dispensary with a waiting-room attached. A staircase, entirely separated from the out-patient department, leads up to an isolation ward, a nurses' room, and a small pantry.

There is also a mortuary, with a separate post-mortem room, situated in the grounds.

